# Differences in peripheral neuropathy in xeroderma pigmentosum complementation groups A and D as evaluated by nerve conduction studies

**DOI:** 10.1186/s12883-021-02414-2

**Published:** 2021-10-09

**Authors:** Tanya J. Lehky, Paul Sackstein, Deborah Tamura, Martha Quezado, Tianxia Wu, Sikandar G. Khan, Nicholas J. Patronas, Edythe Wiggs, Carmen C. Brewer, John J. DiGiovanna, Kenneth H. Kraemer

**Affiliations:** 1 EMG Section, NINDS, NIH, Bethesda, MD USA; 2 Laboratory of Cancer Biology and Genetics NCI, NIH, Bethesda, MD USA; 3 Medstar Georgetown University Hospital, Washington, DC, USA; 4 Laboratory of Pathology, NCI, NIH, Bethesda, MD USA; 5 Clinical Trials Unit, NINDS, NIH, Bethesda, MD USA; 6 Radiology and Imaging Sciences, CC, NIH, Bethesda, MD USA; 7 Otolaryngology Branch, NIDCD, NIH, Bethesda, MD USA

**Keywords:** Xeroderma pigmentosum, Peripheral neuropathy, Neurodegeneration, Sensorineural hearing loss, DNA repair

## Abstract

**Background:**

Xeroderma pigmentosum (XP) is a rare autosomal recessive genetic disorder with defective DNA nucleotide excision repair and associated with a high frequency of skin cancer. Approximately 25% of patients develop progressive neurological degeneration. Complementation groups XP-A and XP-D are most frequently associated with neurological disorders.

**Design/methods:**

This is a retrospective review of patients with XP who were evaluated at NIH from 1986 to 2015 and had nerve conduction studies (NCS). In the complementation groups with peripheral neuropathy, further comparisons of the NCS were made with audiological, brain imaging, neuropsychological assessments that were also performed on most of the patients. Limited neuropathology of XP-A and XP-D patients were examined..

**Results:**

The 33 patients had NCS: XP-A (9 patients), XP-C (7 patients), XP-D (10 patients), XP-E (1 patient), XP-V (4 patients), and XP-unknown (2 patients). Peripheral neuropathy based on nerve conduction studies was documented only in two complementation groups: 78% (7/9) of XP-A patients had a sensorimotor neuropathy while 50% (5/10) of XP-D patients had a sensory neuropathy only. Analysis of sural sensory nerve amplitude in both complementation groups XP-A and XP-D correlated with sensorineural hearing loss (SNHL), MRI/CT severity, and Full-scale Intelligence Quotient (IQ). Analysis of fibular motor nerve amplitude in complementation XP-A correlated with SNHL and MRI/CT severity. Limited follow-up studies showed gradual loss of NCS responses compared to an earlier and more rapid progression of the hearing loss.

**Conclusions:**

Despite similar brain imaging and audiological findings patients, XP-A and XP-D complementation groups differ in the type of neuropathy, sensorimotor versus sensory alone. A few cases suggest that sensorineural hearing loss may precede abnormal NCS in XP and therefore serve as valuable clinical indicators of XP patients that will later develop peripheral neuropathy.

## Background

Xeroderma pigmentosum (XP) is a rare autosomal recessive disorder involving defective DNA nucleotide excision repair (NER) that is characterized by extreme sensitivity to the damaging effects of ultraviolet radiation (UV) resulting in a greater than 2000-fold increase in the frequency of melanoma and non-melanoma skin cancers. Furthermore, approximately 25% of XP patients develop a progressive neurodegeneration [1, 2]. XP is classified into 8 different complementation groups, denoted as XP A-G based on defects in DNA nucleotide excision repair (NER) genes and XP-Variant (XP-V) that has a defect in the polymerase *eta* gene [3]. Patients in complementation groups XP-A and XP-D have the highest frequency of neurological complications along with the greatest photosensitivity or burning on minimal sun exposure [4–6]. A four-decade longitudinal study on the natural history of XP since 1971 showed that the leading cause of death in XP patients was skin cancer. However, neurological degeneration was also a major cause of death [1]. Brain atrophy, cerebellar and basal ganglia degeneration leading to ataxia and severe cognitive decline have all been observed in XP-A and XP-D patients [7, 8]. In conjunction with central nervous system (CNS) deterioration, these patients have been observed to become areflexic and develop an axonal polyneuropathy as well as sensorineural hearing loss [9]. Though visual loss occurs in XP, it is usually the result of direct UV-exposure of the anterior components of the eye rather than specific optic nerve deterioration [10].

The frequency of peripheral neuropathy (PN) and its association with CNS degeneration, in different XP complementation groups remains unknown. In this study, we performed a retrospective review of nerve conduction studies of XP patients examined at NIH over a 30 year period to assess the frequency and type of peripheral neuropathy among patients in different XP complementation groups. In the complementation groups associated with peripheral neuropathy, we further determined the correlation between PN and sensorineural hearing loss (SNHL) as well as markers of CNS deterioration in XP including brain atrophy and loss of cognition.

## Materials and methods

### Patients

Patients were evaluated under clinical protocols, NCT00001813 and NCT00046189, approved by the National Institutes of Health (NIH) Institutional Review Boards. Written, informed consent and assent, was obtained from all patients. The patients underwent multimodality assessment including clinical [11], genetic, MRI/CT imaging, neuropsychological, laboratory and electrodiagnostic evaluation. Records of deep tendon reflexes were available on most of the subjects evaluated. Absence of deep tendon reflexes were considered to be a clinical manifestation of peripheral neuropathy. Available data was retrieved by retrospective review of charts of patients with a clinically confirmed diagnosis of XP at the NIH from 1986 to 2018. Subjects were excluded from the study if no or incomplete nerve conduction studies were performed, if they had a complex diagnosis of XP combined with another disease including XP-trichodystrophy or XP-Cockayne syndrome, or had other comorbidities such as chemotherapy or diabetes. The neurologist, audiologist, neuropsychologist, and neuropathologist who performed the assessments were not blinded to the diagnoses. For clarity in this paper, complementation group A subjects were designated XPA1–9 and complementation group D were designated XPD 1–10 but have also included the prior protocol designated subject numbers in the tables. The following subjects have been previously reported in other articles, XPA3 (XP19BE) [5], XPA5 (XP12BE) [4, 5, 9, 12], XPA6 (XP360BE) [13], XPD1 (XP29BE) [5, 14, 15], and XPD4 (XP33BE) [5].

### Clinical neurophysiology studies

Nerve conduction studies were performed using standard methodology on a Nicolet Viking Select equipment (Natus, Middleton, WI) for the studies performed after 2004 and were compared to department-based normative values. All prior studies used earlier versions of the Nicolet Viking equipment. In most patients, the median and sural sensory nerves and the median and fibular motor nerves were tested although other nerves were selected for clinical indications. No needle Electromyography (EMG) studies were performed. For consistency, the studies were reviewed by one neurologist (TJL). For this EMG lab, the normal nerve conduction study amplitudes are: sural sensory nerve ≥6 μV, median sensory nerve ≥15 μV, fibular motor nerve ≥2.5 mV, and median motor nerve ≥4.5 mV. Though some patients were severely affected by skin manifestations of xeroderma pigmentosum, we did not encounter technical difficulties in obtaining nerve conduction studies.

The presence of a sensory polyneuropathy is defined as diminished or absent sensory nerve amplitudes and minimal to no changes in conduction velocity with preserved motor nerve conduction studies [16]. The presence of a sensorimotor polyneuropathy is defined as a combination of diminished or absent amplitudes in both motor and sensory nerves with minimal to no changes in conduction velocity [16]. In length-dependent neuropathies, the sural sensory nerve and fibular motor nerve studies are predominantly affected.

### Audiological evaluations

Audiologic testing included pure-tone air-conduction (0.25–8 kHz) and bone-conduction (0.25–4 kHz) thresholds and speech audiometry using clinical audiometers in double-walled sound suites that met American National Standards Institute criteria. Audiology results were presented as the four frequency (0.5/1/2/4-kHz)-pure-tone average (4F-PTA), using the right ear results only. Classification of the hearing loss is: < 20 dB hearing level (dBHL) = normal hearing, > 20–40 dBHL = mild hearing loss, > 40–70 dBHL = moderate hearing loss, > 70–95 = severe hearing loss, > 95 profound hearing loss. For consistency, the studies were reviewed by one audiologist (CCB).

### Brain imaging

MRI or CT imaging of brain was performed at NIH or reviewed from outside sources. Cerebral and cerebellar atrophy were classified on a four-point scale: normal (0), mild (1), moderate (2), or severe (3) depending on the extent of volume loss at each patient’s age. For consistency, the studies were reviewed and graded by one neuroradiologist (NP).

### Neuropsychological studies

Wechsler scales appropriate for age were performed to yield a Full-Scale Intelligence Quotient (IQ). Those XP patients unable to do the Wechsler test because of limited cognitive ability or very young age, were excluded. For the Full-Scale IQ, a score of less than 80 was considered to be abnormal. For consistency, the studies were reviewed by one neuropsychologist (EW).

### Neuropathology

Pathology slides from autopsy were available for one XP-A patient (XPA5) and one XP-D patient (not evaluated by NCS) for review. These autopsy reports had been previously published [4], and available specimens of muscle and nerve were reexamined. New hematoxylin & eosin (H&E) stained sections of peripheral nerve and muscle were prepared from formalin-fixed paraffin embedded material. For consistency, the studies were reviewed by one neuropathologist (MQ).

### Data analysis

Descriptive statistics of demographic data were presented in table and two-sample t-test was used to evaluate the difference in age between XP-A and XP-D group For each of neurophysiological measures of motor and sensory amplitudes, two-sample t-test was used to evaluate the difference between XP-A and XP-D group, and Pearson correlation analysis was used to evaluate the association with hearing level. The correlation analysis was performed for XP-A and XP-D combined or separately depending on the test of homogeneity of regression slopes (or interaction between XP group and hearing level) by analysis of covariance (ANCOVA). The correlation analysis would be performed separately if the interaction was significant (*p* < 0.05), because the significant interaction indicated that the linear relationship between the neurophysiological measures clinical outcome and hearing level in XP-A group was different from that in XP-D. Normality assumption for two-sample t-test and ANCOVA was examined using Shapiro-Wilk test.

### Data availability policy

Anonymized data will be shared by request from any qualified investigator.

## Results

### Demographics

**Fig. 1:**
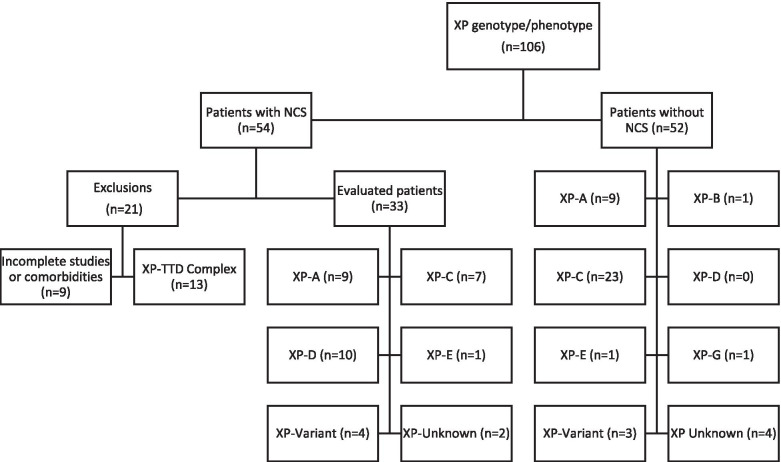
Algorithm of Subjects Studied. Of the 106 subjects with an XP genotype/phenotype, 54 subjects had nerve conduction studies and 33 subjects were included in the final evaluation. NCS – nerve conduction studies, XP – xeroderma pigmentosum

For the study, 106 patients had been evaluated for XP phenotype/genotype over 30 years (Fig. 1) with electrodiagnostic studies performed on 54 of the patients. The majority of the electrodiagnostic studies were performed between 2004 and 2015, though 7 patients had studies performed between 1986 and 1993. Twenty-one patients were excluded from final analysis: 12 patients with XP-TTD complex [1] and 9 patients were excluded because of confounding comorbidities or incomplete studies, including 6 XP-C patients, 1 XP-D patient, 1 XP-E patient and 1 XP-G patient. In the XP-D complementation group, the one excluded patient had limb girdle muscular dystrophy. Of the remaining 33 patients (Table 1), 9 (27%) patients were in complementation group XP-A, 7 (21%) patients were in XP-C, 10 (30%) patients were in XP-D, 1 (3%) was in XP-E, 4 (12%) were in XP-V, and 2 (6%) had an unknown mutation causing XP (Table 1). The total group demographics were 14 males and 19 females with a mean age at initial NCS study was 20.7 ± 13.6 years (range 3–54 years).

**Table 1:** Demographics of XP Patients with Nerve Conduction Studies

	Complementation Group					
	XP-A	XP-C	XP-D	XP-E	XP-V	XP unk
Number	9	7	10	1	4	2
Mean Age ± Std Dev	16.7 ± 9.0	13.1 ± 6.9	18.5 ± 2.1	42	43.5 ± 9.1	12, 36
(Range)	(3–24)	(5–25)	(5–43)		(34–54)	
Sex (F:M)	5:4	4:3	4:6	1:0	3:1	1:1
Abn SNCS	7/9	0/7	5/10	0/1	0/4	0/2
Abn MNCS	7/9	0/7	0/10	0/1	0/4	0/2
Abn Hearing	6/8	0/6	5/10	0/1	0/3	0/1
Abn MRI/CT	7/8	0/4	6/8			0/1
Abn IQ	5/6	0/4	3/6			0/1

*Abbreviations*: *XP-A* Xeroderma pigmentosum complementation group A, *XP-C* Xeroderma pigmentosum complementation group C, *XP-D* Xeroderma pigmentosum complementation group D, *XP-E* Xeroderma pigmentosum complementation group E, *XP-V* Xeroderma pigmentosum variant, *XP unk* Xeroderma pigmentosum – Unknown mutation, *F* Female, *M* Male, *Abn* Abnormal, *SNCS* Sensory nerve conduction studies, *MNCS* Motor nerve conduction studies (fibular nerve), *MRI* Magnetic resonance imaging, *CT* Computed tomography, *IQ* Intelligence quotient, *Std dev* Standard deviation

The XP-A and XP-D complementation groups were the only groups that had evidence for both peripheral neuropathy and other neurological disorders of hearing loss, brain imaging abnormalities, and abnormal IQ (Table 1). Therefore, we limited the remainder of the analysis to these two groups including the correlation analysis, long-term follow-up studies and pathology. These two complementation groups were slightly younger than the total group of patients in Table 1, but there was no significant difference in age (*p* = 0.48) between the two groups (mean ± standard deviation, range; XP-A -18.5 ± 2.1, 3–24, XP-D - 16.7 ± 9.0, 5–43). Follow-up studies were available for six XP-A and XP-D patients and limited available neuropathology was reviewed.

### XP-A findings

Seven of the nine (78%) XP-A patients had evidence for an axonal sensorimotor polyneuropathy based on nerve conduction studies with the findings summarized in Tables 1, and 2. For the seven XP-A patients with abnormal nerve conduction studies, the median sensory nerve had an amplitude of 4.62 ± 4.38 μV with conduction velocity 47 ± 6 m/s; the sural sensory nerve had an amplitude of 0.86 ± 1.46 μV with conduction velocity 47 ± 6 m/s, the median motor nerve had an amplitude 9.35 ± 1.62 mV with a conduction velocity 46 ± 3 m/s, and the fibular motor nerve had an amplitude of 1 ± 1 mV amplitude with a conduction velocity of 33 ± 6 m/s.. For the two XP-A patients with normal nerve conduction studies, the median sensory nerve had amplitudes of 37 μV and 33 μV with conduction velocities of 58 and 56 m/s, the sural sensory nerve had both amplitudes of 10 μV with a conduction velocity of 52 m/s and 43 m/s, the median motor nerve had amplitudes of 10.4 and 5.6 mV with conduction velocities of 57 m/s and 62 m/s, and the fibular motor nerve had amplitudes of 4.7 mV and 5.7 mV with both conduction velocities of 49 m/s. The 4F-PTA showed that six of these patients (XPA1–6) also had mild to severe sensorineural hearing loss and MRI/CT imaging abnormalities. One subject (XPA-7) with neuropathy, a 3 year old, did not have audiology testing. MRI/CT imaging findings observed in the same subjects with neuropathy included cerebral atrophy, cerebellar atrophy and in 2 pediatric patients, hypomyelination was noted. Six XP-A patients had Full-Scale IQ testing and all were abnormal, regardless of the presence or absence of neuropathy. XP-A patients who had neuropathy, expectedly, also had absent DTRs. However, one XP-A patient (XPA-9) eventually lost her DTRs though her NCS remained normal. Information on other facets of the neurological exam were limited by the cognitive status of the patients.

**Table 2:** XP-A Patient Findings

Patient	Age at Test Date	Sex	Neuropathy		Audiology	MRI/CT Imaging		IQ Testing		DTR
ID # (Protocol designated ID)			Abn SNC	Abn MNC	SNHL	Cerebral Atrophy	Cerebellar Atrophy	Hypo- myelination	IQ < 80	Absent
XPA1 (XP81BE)	18	F	+	+	Mod	1	1	–	+	+
XPA2 (XP79BE)	24	M	+	+	Mild	2	1	–	+	+
XPA3 (XP19BE)^a^	33	M	+	+	Mod	2	2	–	+	+
XPA4 (XP460BE)	9	F	+	+	ND	1	1	+	ND	+
XPA5 (XP12BE)^a-d^	19	F	+	+	Severe	3	2	–	+	+
XPA6 (XP360BE)^e^	10	M	+	+	Mod	2	1	+	ND	+
XPA7 (XP53BE)	3	F	+	+	ND	1	0	–	ND	–
XPA8 (XP337BE)	21	M	–	–	Normal	0	0	–	+	–
XPA9 (XP631BE)	13	F	–	–	Normal	0	0	–	+	–

*Abbreviations*: *XPA* Xeroderma pigmentosum – A, *F* Female, *M* Male, *Abn* Abnormal, *SNC* Sensory nerve conduction studies, *MNC* Motor nerve conduction studies (fibular nerve), *MRI* Magnetic resonance imaging, *CT* Computed tomography, *IQ* Intelligence quotient, *DTR* Deep tendon reflexes, *ND* Not done, *Mod* Moderate

Footnotes: ^a^ Totonchy MB,et al. 2013 (Ref. [5]), ^b^ Lai JP, et al. 2013 (Ref. [4]), ^c^ Viana LM, et al. 2013 (Ref. [9]), ^d^ Ramkumar HL, et al. 2011 (Ref. [12]), ^e^ Christen-Zaech S, et al. 2009 (Ref. [13])

### XP-D findings

In the XP-D patients, 5 patients of 10 studied (XPD1–5) or 50% had evidence for an axonal sensory neuropathy based on nerve conduction studies with the findings summarized in Tables 1, and 3. For the 5 XP-D patients with abnormal nerve conduction studies, the median sensory nerve had an amplitude 5.20 ± 2.68 μV with a conduction velocity 49 ± 5 m/s, the sural sensory nerve had an amplitude of 1.75 ± 1.70 μV with a conduction velocity 46 ± 4 m/s, the median motor nerve had an amplitude 9.64 ± 4.54 mV with a conduction velocity 55 ± 3 m/s, and the fibular motor nerve had an amplitude of 3.62 ± 1.15 mV with a conduction velocity of 42 ± 2 m/s.. For the 5 XP-D patients with normal nerve conduction studies, the median sensory nerve had an amplitude 32.6 ± 8.26 μV with a conduction velocity 54 ± 5 m/s, the sural sensory nerve had an amplitude of 13.2 ± 6.53 μV with a conduction velocity 48 ± 5 m/s, the median motor nerve had an amplitude 11.32 ± 1.14 mV with a conduction velocity 57 ± 5 m/s, the fibular motor nerve had an amplitude of 5.4 ± 1.54 mV with a conduction velocity of 48 ± 4 m/s. These patients (XPD1–5) also had mild to moderate sensorineural hearing loss and abnormal MRI/CT images of the brain. The MRI/CT findings included varying degrees of cerebral and/or cerebellar atrophy. XP-D patients without neuropathy had normal hearing and brain imaging, though one patient (XPD6) showed development of cerebral atrophy between ages 9 and 14. Low Full-Scale IQs were observed in all patients with neuropathy and in two patients without neuropathy. All XP-D patients with peripheral neuropathy had absent DTRs while only one patient without peripheral neuropathy (XPD6) had absent DTRs. This was the same patient noted to be developing recent signs of brain atrophy in brain imaging. Clinical exams of the 5 patients with neuropathy noted decreased distal sensation without weakness.

**Table 3:** XP-D Patient Findings

Patient	Age at Test Date	Sex	Neuropathy		Audiology	MRI/CT Imaging			IQ Testing	DTR
ID # (Protocol designated ID)			Abn SNC	Abn MNC	SNHL	Cerebral Atrophy	Cerebellar Atrophy	Hypo- myelination	IQ < 80	Absent
XPD1 (XP29BE)^a-c^	22	M	+	–	Mod	3	2	–	+	+
XPD2 (XP528BE)	19	M	+	–	Mild	1	0	–	+	+
XPD3 (XP32BE)	42	M	+	–	Mild	2	1	ND	ND	+
XPD4 (XP33BE)^a^	31	F	+	–	Mod	2	1	–	+	+
XPD5 (XP400BE)	17	M	+	–	Mild	1	0	–	+	+
XPD6 (XP116BE)	5	F	–	–	Normal	0	0	–	–	+
XPD7 (XP82BE)	11	F	–	–	Normal	0	0	–	–	ND
XPD8 (XP30BR-D)	13	M	–	–	Normal	0	0	–	–	ND
XPD9 (XP341BE)	3	M	–	–	Normal	0	0	–	+	_
XPD10 (XP416BE)	13	F	–	–	Normal	0	0	–	+	–

*Abbreviations*: *XPD* Xeroderma pigmentosum – D, *F* Female, *M* Male, *Abn* Abnormal, *SNC* Sensory nerve conduction studies, *MNC* Motor nerve conduction studies (fibular nerve), *MRI* Magnetic resonance imaging, *CT* Computed tomography, *IQ* Intelligence quotient, *DTR* Deep tendon reflexes, *ND* Not done, *Mod* Moderate

*Footnotes*: ^a^ Totonchy MB, et al. 2013 (Ref. [5]), ^b^ Ueda T, et al. 2009 (Ref. [14]), ^c^ Zhou X, et al. 2013 (Ref. [15])

### Comparison of XP-A and XP-D complementation groups

Comparison of the nerve conduction studies between the XP-A and XP-D complementation groups, using a two-sample t-test, showed a significant difference in the fibular motor amplitudes (*p* = 0.0208, for XP-A: mean = 2.15, SD = 2.01, median = 1.85, 95%CI = 0.47–3.83; for XP-D: mean = 4.47, SD = 1.82, median = 3.8, 95%CI = 3.16–5.78; effect size or Cohen’s d = 1.22) but not the sural sensory, median sensory and median motor amplitudes. There was no significant difference in the hearing loss, cortical atrophy on MRI/CT imaging and Full-scale IQ between the two complementation groups.

### Association evaluations (XP-A and XP-D)

**Fig. 2:**
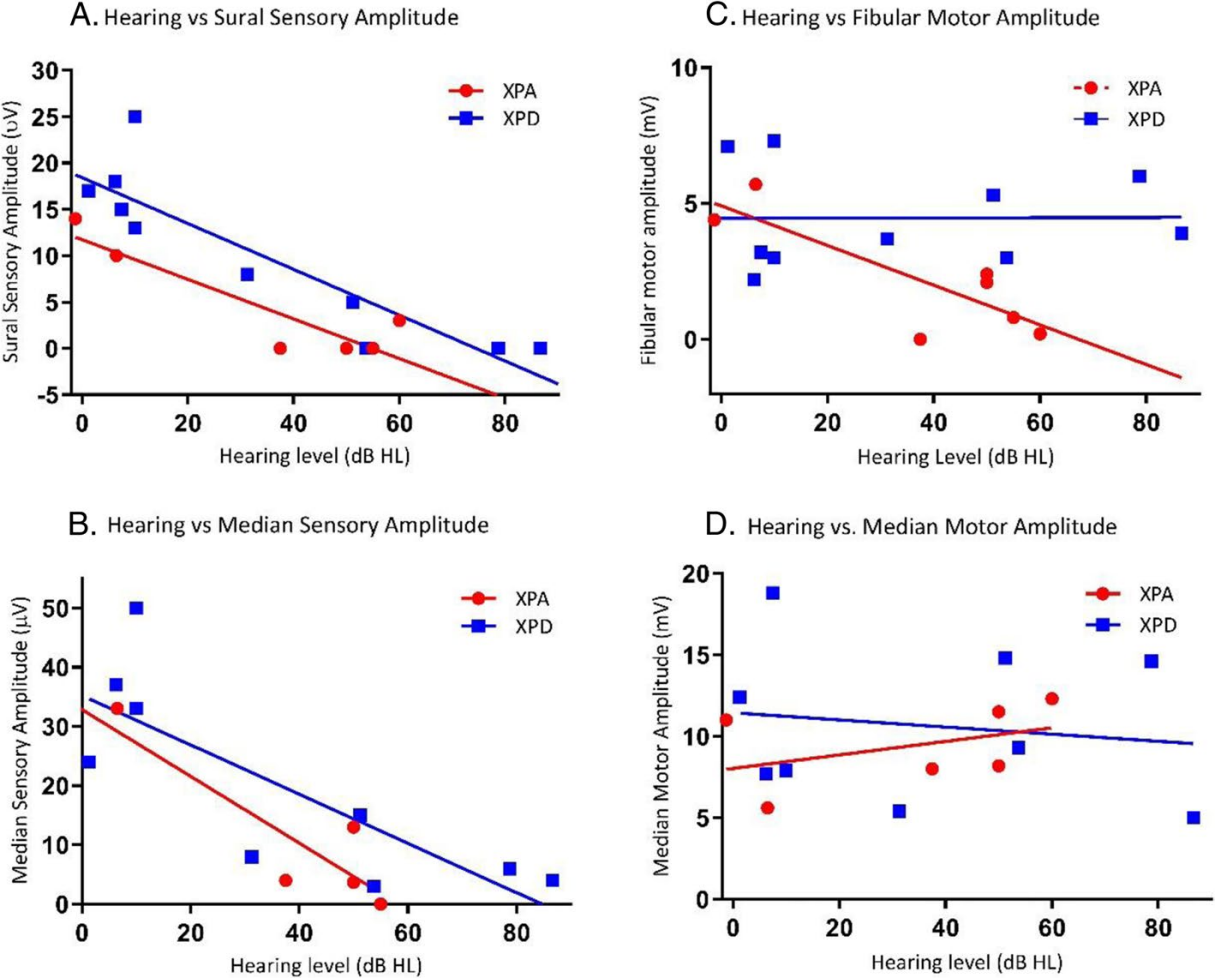
Hearing loss vs nerve conduction studies in XP-A and XP-D. The right ear 4F-PTA, defined as the four frequency (0.5/1/2/4-kHz) pure tone average, was plotted against the amplitudes of the following: **A** Sural sensory nerve, **B** Median sensory nerve, **C** Fibular motor nerve, **D** Median motor nerve. ANCOVA was used to study the interaction between complementation group and hearing level. Normal hearing: 4F-PTA ≤ 20 dB HL. Normal NCS findings: sural sensory ≥6 μV, median sensory ≥15 μV, fibular motor ≥2.5 mV, median motor ≥4.5 mV

For sural sensory amplitude, the Pearson correlation analysis using a combined XP-A and XP-D group found significant linear correlation with hearing loss (r = − 0.845, R^2^ = 0.714, *p* < 0.001, Fig. 2a), MRI/CT severity (r = − 0.796, R^2^ = 0.634, p < 0.001), and Full-Scale IQ (r = 0.520, R^2^ = 0.27, *p* = 0.047) reflecting deterioration reflecting deterioration of sensory amplitudes with hearing loss, MRI/CT severity and Full-scale IQ.

For median sensory amplitude, significant linear correlation was also found with hearing loss (r = 0.842, R2 = 0.709, *p* < 0.001, Fig. 2b) and MRI/CT severity (r = 0.865, R^2^ = 0.748, *p* < 0.001) but not with IQ (*p* = 0.258).

For fibular motor amplitude (Fig. 2c), the linear correlation with hearing was significant (r = − 0.828, R^2^ = 0.686, *p* = 0.022) in XP-A group, but not in XP-D group (*p* = 0.985). This was in keeping with the sparing of motor nerve abnormalities in XP-D group. The linear correlation with MRI/CT severity was significant (r = − 0.761, R^2^ = 0.58, *p* = 0.0285) in XP-A group, which was similar to the sural sensory amplitude, but not significant (*p* = 0.632) in XP-D group. The correlation with Full-scale IQ was not significant in either XP-A (*p* = 0.627) or XP-D (*p* = 0.689).

For median motor amplitude (Fig. 2d), correlation coefficients were not significant for hearing, MRI/CT severity, or Full-Scale IQ.

For these analyses, the correlation coefficients of the amplitude variable with each of the clinical variables were estimated based on combined or separate XP-A and XP-D groups depending on the significance of the interaction between XP group and clinical variables, and the significance of the differences of variables between XP-A and XP-D groups. All amplitude variables except for fibular motor amplitude variable, the correlation analyses were performed by combining XP-A and XP-D groups, since the interaction between XP group (XP-A vs. XP-D) and each of the three clinical variables, hearing loss, MRI/CT severity and IQ, was not significant (p > = 0.230) in ANCOVA and the differences between XP-A and XP-D group in any of the three sensory amplitude variables and three clinical variables were not significant in t-test. For fibular motor amplitude variable, the correlation analysis was performed for XP-A and XP-D group separately, since the interaction between XP group and hearing loss was significant (*p* = 0.050, Fig. 2c), and the difference in fibular motor amplitude between XP-A and XP-D groups was significant (*p* = 0.0208).

### Follow-up evaluations

**Fig. 3:**
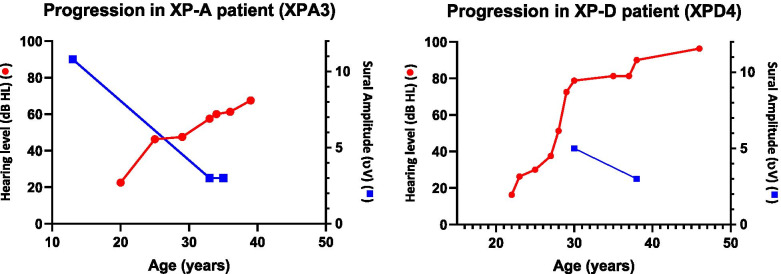
Follow-up hearing and sural sensory nerve studies. **A** Patient XPA3 (neurological involvement) **B** Patient XPD4 (neurological involvement). Age of patient (X axis) plotted versus hearing (dB HL) (left Y axis) and sural amplitude (μV) (right Y axis). Normal hearing:4F-PTA ≤ 20 dB HL. Normal NCS findings: sural sensory ≥6 μV, fibular motor ≥2.5 mV

Follow-up evaluations were available on two XP-A and four XP-D patients. Patient XPA3 (XP19BE) was evaluated at age 14, 35, and 37 years and had neurologic deterioration during this time (Fig. 3a). Sural sensory nerve amplitude decreased from 10.8 μV to 3 μV over the first 19 years but did not change any further when retested 2 years later. The initial study was performed on an older EMG machine which may have affected the results. Sequential nerve conduction studies of lower extremity motor nerves were not available. Patient XPA8 (XP337BE) did not have overt signs of neurologic deterioration and motor and sensory NCS at age 14 and 23 years have remained stable.

In the XP-D complementation group, the two patients, XPD1 (XP29BE) and XPD4 (XP33BE) with peripheral neuropathy (XPD-1 and XPD-4) and neurological deterioration showed a minimal decline in the sensory responses with stable motor responses (Fig. 3b). Patient XPD6 (XP116BE), an initially neurologically normal patient with recent signs of cerebral atrophy on CT brain images, had a 50% decrease in sural sensory response (25 μV to 12 μV) over 4 years. Patient XPD9 (XP341BE), also neurologically normal, showed no change in the median and sural sensory nerve amplitudes in a three-year follow-up period. There were no decreases in the motor responses for any of the XPD patients.

### Neuropathology

Nerve and muscle pathology was compared between the XP-A and XP-D patient (Fig. 3) and previously reported [4]. XPA5 (XP12BE) patient died at age 44, was cachectic (< 3%tile wt.), nonambulatory, with no detectable DTRs, and had hearing loss and optic atrophy [5]. The XP-D patient (XP18BE) died at age 45 and was not cachectic (50% tile wt.) but was nonambulatory, with no detectible DTRs, and had hearing loss [5]. There were no available nerve conduction studies for this XP-D patient. Both patients had had adequate nutrition maintained through g-tubes.

Sampling from psoas muscle and median and vagus nerve were formalin-fixed, paraffin embedded and slides were stained with hematoxylin and eosin (H&E) for routine light microscopy evaluation. For electron microscopy studies, samples were fixed in glutaraldehyde. Limited immunohistochemical stains for myofibers.

were performed on the XP-A patient. Other preparation including enzyme histochemistry and teased fibers for muscle and nerve evaluation were not available.

**Fig. 4:**
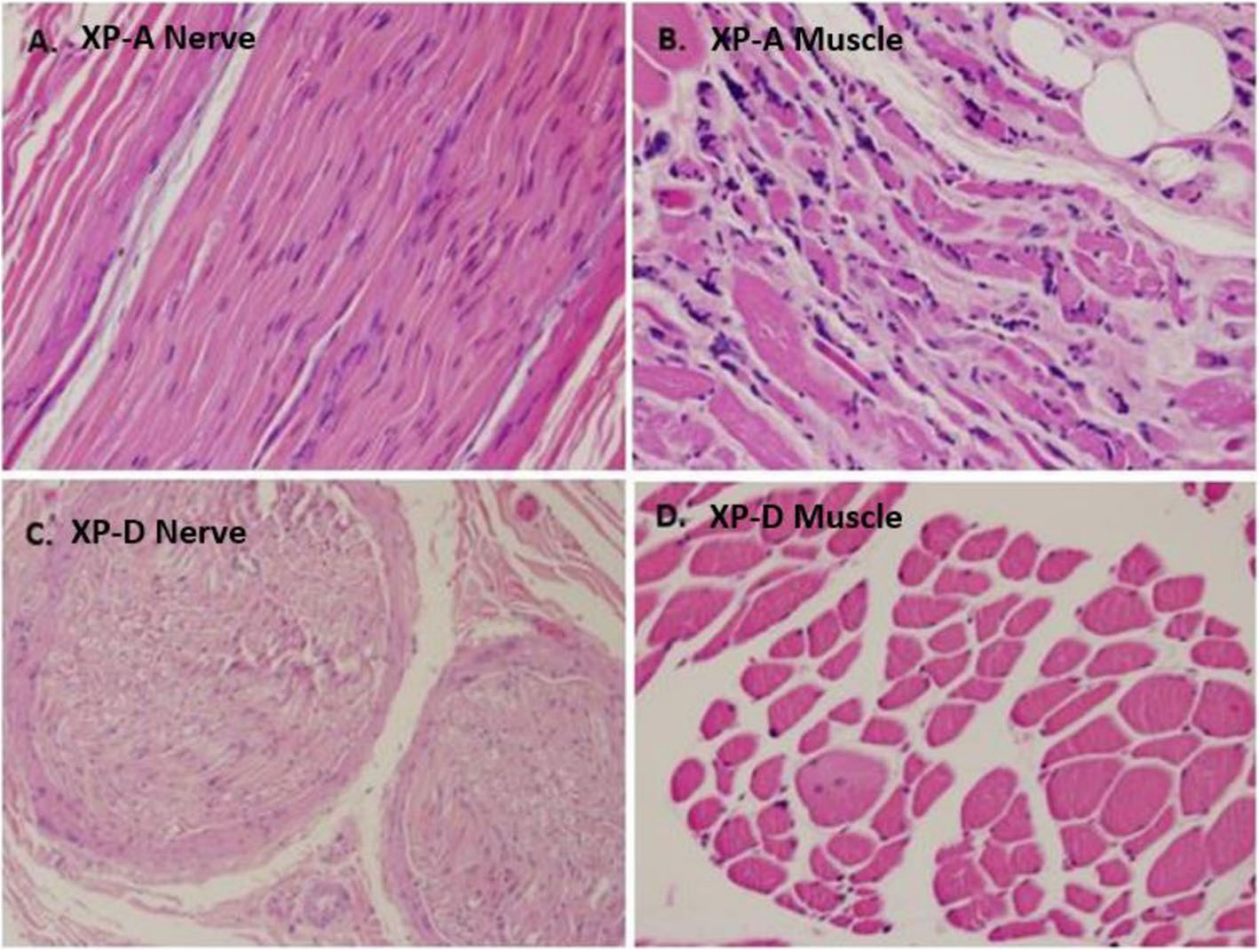
Nerve and muscle pathology of XP-A and XP-D patients. **A** XPA (XP5 - XP12BE) patient median nerve - H&E, 20x, normal nerve architecture on longitudinal section. **B** XP-A (XP5 - XP12BE) patient psoas muscle – H&E, 20x, marked variation in myofiber size characterized by the presence of many rounded and angular atrophic fibers, approaching panfascicular atrophy in some areas with fatty infiltration. Numerous pyknotic nuclear clumps are also present. **C** XP-D patient nerve from arm but not specified – H&E, 10x, normal nerve architecture on cross-section. **D** XP-D patient psoas muscle – H&E, 20x, mild variation of muscle fiber size with occasional small-sized angulated fibers. (Modified from JP Lai, et al. 2013, permission by Dr. K. Kraemer)

In the XP-A patient, electron microscopy of the vagus nerve showed axonal degeneration and degenerative changes of the myelin sheath consistent with an axonal neuropathy (not shown) though median nerve only showed mild loss of large myelinated axons (Fig. 4A). The psoas muscle (Fig. 4B) showed findings characteristic of chronic denervation atrophy with many angular atrophic fibers, myofiber type-grouping, pyknotic nuclear clumps and fat infiltration. There was no evidence of myopathic type changes, inflammation or vasculitis. Slow and fast myosin antibody stains showed nonselective atrophy with evidence of myofiber-type grouping.

In the XP-D patient, the nonspecified nerve from the arm under light microscopy had normal architecture (Fig. 4C) as well as normal pathology of a dorsal root ganglion (not shown). There were mild neurogenic findings on psoas muscle pathology (Fig. 4D).

## Discussion

Consistent with observations in past studies, patients with mutations in XP-A and XP-D develop neurological complications including peripheral neuropathy, brain atrophy, hearing loss, and low IQ. These neurological findings are generally not found in patients with mutations in XP-C, XP-E or XP-V. Though there were a limited number of patients in each group, our electrodiagnostic findings suggests that the peripheral nerve manifestations differ between these two complementation groups, with the XP-A patients having a sensorimotor neuropathy and XP-D patients having only a sensory neuropathy. Sensorimotor neuropathy has been reported in XP-A patients [17, 18] as well as the associated loss of deep tendon reflexes [19] though selectivity of XP-D neuropathies to the sensory nerve has not been previously described. In both XP-A and XP-D complementation groups, the peripheral neuropathy becomes evident within the first or second decade and appears to progress slowly as shown by the gradual decline in sural sensory responses in the follow-up studies.

We also demonstrated that there were correlations between the sural sensory amplitude and the other three markers of neurodegeneration: hearing loss, cortical atrophy on brain imaging and low IQ. This suggests that the neurodegeneration is affecting the different parts of the nervous system may be occurring concurrently. The correlations for the fibular motor amplitude were more limited, reflecting the differences between the XP-A and XP-D neuropathy presentations. The relative sparing of the median motor nerve in XP-A suggests that there is likely a length-dependent motor nerve degeneration. The neuropathology of the PNS of the XP-A patient shows an axonal neuropathy associated with chronic neurogenic denervation of the muscles consistent with the sensorimotor neuropathy noted on electrodiagnostic studies. This finding was not observed in the neuropathology of the XPD patient.

In XP, perturbations in the NER pathway result in helix-distorting lesions in the DNA which leads to various end organ damage [20–24]. Neuronal and glial cell injury, both in mature nonreplicating cells and immature replicating cells, are subject to oxidative stress which is normally repaired by a host of DNA repair mechanisms [25]. The XPC and XPE proteins are responsible for damage recognition, the XPA protein is involved in the verification and complex formation at the site of DNA damage. The XPD protein is one of the helicases involved in unwinding the DNA prior to excision and repair. The other XP gene products are involved in DNA complex stabilization, DNA cleavage, and finally repair of the DNA strand with polymerases and ligases. Clearly, the integrity of the NER pathway and the XP gene complementation groups is crucial for maintenance of DNA repair in the central and peripheral nervous systems though the exact mechanism is not known. Since neuronal cells are not predisposed directly to the injurious effects of ultraviolet (UV) radiation, other mechanisms for neuronal injury in XP patients have been proposed [26]. One suggested mechanism may be generation of oxidative stress [27]. The reactive oxygen species (ROS) are constantly generated during aerobic metabolism. One of the ROS is hydroxyl radical, the key endogenous source of DNA damage. Hydroxyl radicals induce many different base lesions including cyclopurine deoxynucleosides (CyPU) that are known to be removed by the NER pathway [28–31]. Accumulation of cyPU in neuronal cells with inability to undergo DNA repair may lead to neurodegeneration [32]. Other suggested mechanisms may be mitochondrial dysfunction and role of these proteins in non-DNA repair pathways [26, 33]. Generation of neural stem cells and post-mitotic neurons from XP-A patient induced pluripotent stem cells (IPSCs) showed hypersensitivity to DNA damage-induced apoptosis following UV exposure [34]. The neurophysiological findings in this paper suggests that there may be slightly different mechanisms of neurodegeneration associated with XP-A and XP-D complementation groups that, at least in the peripheral nervous system, result in two types of neuropathy. Better understanding of pathways may be beneficial in designing clinical studies that address neurotoxicities in XP-A and XP-D since controlled clinical studies in this area have been limited to date [35, 36]. There is no known prevention or treatment of the neurological sequelae of XP though antioxidant therapy, upregulation of autophagy, sulfonylureas, and nicotinamide have been considered possible avenues of treatment [35, 37].

Because XP is a rare disease, the number of subjects in each complementation group is small and the robustness of our findings is limited. However, it supports the importance of DNA NER in the survival of central and peripheral neurons with defects in the pathway resulting in variable forms of peripheral nerve degeneration. Continued studies on the peripheral nerve manifestations of XP-A and XP-D may help to further elucidate the role of the XP gene defects in the pathogenesis of neuropathy, particularly as related to the DNA NER pathway. Also, it would be important to expand the evaluation of peripheral nervous system deterioration by including investigation of small fiber neuropathies in the XP complementation groups and to determine if there is electrophysiological evidence of anterior horn motor neuron deterioration, particularly in the XP-A patients.

## Conclusions

The findings in this study are notable for the distinction between XP-A and XP-D on the basis of the type of peripheral neuropathy. CNS deterioration and hearing loss does not appear to be different between the two XP complementation groups. In clinical evaluation of XP-A and XP-D patients, it is important to include evaluation of peripheral neuropathy as part of the complete neurological evaluation that also includes hearing testing, brain imaging, and cognitive testing.

## Data Availability

The datasets used and/or analysed during the current study are available from the corresponding author on reasonable request.

## Abbreviations

4F-PTA: Four frequency (0.5/1/2/4-kHz) pure-tone average
CNS: Central nervous system
CT: Computed tomography
dB HL: Decibel hearing level
DTR: Deep tendon reflexes
EMG: Electromyography
H & E: Hematoxylin and eosin
IQ: Intellectual quotient
kHz: Kilohertz
MRI: Magnetic resonance imaging
μV: Microvolt
mV: Millivolt
NER: Nucleotide excision repair
NIH: National Institutes of Health
NCS: Nerve conduction studies
PN: Peripheral neuropathy
SNHL: Sensorineural hearing loss
TTD: Trichothiodystrophy
UV: Ultraviolet radiation
XP: Xeroderma pigmentosum
